# Perivascular Space Analysis: A Possible Tool for Predicting Cerebral Amyloid Angiopathy in Pathology‐Confirmed Cases

**DOI:** 10.1002/alz.089886

**Published:** 2025-01-09

**Authors:** Maryam Fotouhi, Nasim Sheikh‐Bahaei, Jeiran Choupan

**Affiliations:** ^1^ USC Mark and Mary Stevens Neuroimaging and Informatics Institute, Keck School of Medicine, University of Southern California, Los Angeles, CA USA; ^2^ Keck School of Medicine, University of Southern California, Los Angeles, CA USA; ^3^ NeuroScope Inc., New York, NY USA

## Abstract

**Background:**

Diagnosis of cerebral amyloid angiopathy (CAA) presents a significant challenge in determining whether to propose anti‐amyloid treatment plans. The identification of perivascular spaces (PVS) through MRI serves as a possible strategy to elucidate the physiopathological interconnections between the brain's clearance mechanisms and the accumulation of amyloid. This study endeavors to the association between PVS morphology and CAA pathology.

**Methods:**

T1‐weighted and T2‐FLAIR MRI data were obtained from the Alzheimer’s Disease Neuroimaging Initiative (ADNI). Demographic details (sex, age, post‐mortem interval) and ADNI pathologic scores were gathered. Histological images provided scores for cerebral amyloid angiopathy. Leveraging our on the last premortem MRI, we automatically mapped regional PVS in white matter and basal ganglia. analysis was then employed to investigate whether premortem regional PVS volume fractions serve as predictors for postmortem CAA pathology, accounting for age, sex, and post‐mortem interval.

**Results:**

The investigation revealed notable predictive associations between specific occipital regions' PVS volume fractions and the presence of amyloid angiopathy pathology, after multiple comparison correction. Specifically, the study identified the right cuneus PVS volume fraction as a robust predictor for the manifestation of amyloid angiopathy pathology (t=3.12, p=0.001). Similarly, the left precuneus PVS volume fraction emerged as a statistically significant predictor (t=2.78, p=0.005).

**Conclusion:**

This study presents a noteworthy advancement in amyloid angiopathy diagnosis, employing standard MRI analysis of perivascular spaces. The identified correlation offers a pragmatic means to enhance diagnostic accuracy without complexity. This research underscores the potential applicability of PVS analysis in routine clinical practice for early CAA detection, contributing significantly to the practical improvement of CAA diagnostics.

**Reference**: 1. Sepehrband, F., Barisano, G., Sheikh‐Bahaei, N. et al. Image processing approaches to enhance perivascular space visibility and quantification using MRI. Sci Rep 9, 12351 (2019). https://doi.org/10.1038/s41598‐019‐48910‐x

